# Quantitative analysis of dinoflagellates and diatoms community via Miseq sequencing of *actin* gene and v9 region of 18S rDNA

**DOI:** 10.1038/srep34709

**Published:** 2016-10-10

**Authors:** Liliang Guo, Zhenghong Sui, Yuan Liu

**Affiliations:** 1 Key Laboratory of Marine Genetics and Breeding of Ministry of Education, College of Marine Life Sciences, Ocean University of China, Qingdao 266003, China; 2 Institution of Plastic Surgery, Weifang Medical University, Weifang, 261042, China

## Abstract

Miseq sequencing and data analysis for the *actin* gene and v9 region of 18S rDNA of 7 simulated samples consisting of different mixture of dinoflagellates and diatoms were carried out. Not all the species were detectable in all the 18S v9 samples, and sequence percent in all the v9 samples were not consistent with the corresponding cell percent which may suggest that 18S rDNA copy number in different cells of these species differed greatly which result in the large deviation of the amplification. And 18S rDNA amplification of the microalgae was prone to be contaminated by fungus. The amplification of *actin* gene all was from the dinoflagellates because of its targeted degenerate primers. All the *actin* sequences of dinoflagellates were detected in the act samples except act4, and sequence percentage of the dinoflagellates in the act samples was not completely consistent with the dinoflagellates percentage of cell samples, but with certain amplification deviations. Indexes of alpha diversity of *actin* gene sequencing may be better reflection of community structure, and beta diversity analysis could cluster the dinoflagellates samples with identical or similar composition together and was distinguishable with blooming simulating samples at the generic level. Hence, *actin* gene was more proper than rDNA as the molecular marker for the community analysis of the dinoflagellates.

Quantitative analysis of phytoplankton community is important for ecological studies and challenging, however, facing up to the problems of low accuracy of species identification which relies heavily on personnel experience, low efficiency and time consuming. To overcome the restriction, many researches were operated to exploit new technique for exactly and quickly quantification of water samples, including which is via high-throughput sequencing of barcoding genes.

It was the first application of high-through sequencing for the species diversity and community structure of marine planktonic microorganism that Amaral-Zettler^1^ designed the universal primers for 18S v9 region and obtained the community structure of the marine microorganisms via 454 pyrosequencing. Yu^2^ studied the nanoplankton diversity and the community structure of different stations in Bohai Bay during the brown tide via 18S v9 region Hiseq sequencing, and found that the blooming was dominated by two brown microalgae. Kermarrec *et al*.^3^ compared the SSU rDNA, *rbcL* and *cox1* amplicons of the freshwater diatoms generated via the method of DNA library construction and 454 pyrosequencing. The result showed that 454 pyrosequencing may reflect more species and taxon units. Mahé *et al*.^4^ operated the 18S v9 region sequencing of the same eukaryotic microbial sample via both 454 pyrosequencing and Miseq sequencing and the result of the analysis showed that both of the methods gave similar diversity and species abundance, however, Miseq revealed more sequences and more taxon because of deeper depth of sequencing.

The studies of analyzing the microbial community structure mentioned above were all based on the high variable region of rDNA sequence. However, the copy number of rDNA greatly varied in different species (prokaryotes and eukaryotes) that might cause large error for species abundance assessment. This problem has been raised for major concerning. Pinto and Raskin^5^ used 16S rDNA sequencing to study the simulated community of bacteria and archaea and found that the error generated from the PCR of mixed DNA was crucial for the accuracy of community structure evaluation. Ye *et al*.^6^ obtained the 16S rDNA sequences of bacterial communities in different sections of a municipal wastewater treatment plant by 454 pyrosequencing, analyzed these community structures and the cell quantity of each species by introduced the rDNA copy number of each species in the bacterial rDNA library, thereby got more precise bacterial community structures. Wu *et al*.^7^ thought it was limited to study the phylogenetic analysis and community structure of multicellular organism by SSU-rDNA sequencing, they also analyzed 40 protein codon gene family (ribosomal protein genes, eukaryotic elongation factor 2, etc.) in different groups and found that some were effective markers for the analysis of phylogenetic and community structure.

Some protein codon genes in nuclear were used to do the phylogenetic analysis and species identification of dinoflagellates, such as heat shock protein 90 gene (HSP90), eukaryotic elongation factor 2 (*elf2*) and *actin* gene. The copy number of these genes was much fewer and stable than that of rDNA. For example, the copy number of *actin*, α-*tubulin* and HSP90 gene in *Oxyrrhis marina* respectively was 33.7, 10.4 and 5.4^8^. Therefore, if housekeeping genes and some other nuclear genes, which have few copies and could distinguish species, were used to build clone library or do high through sequencing, species composition and relative abundance of the environmental samples could be reflected more precisely.

In this report, *actin* gene and 18S v9 rDNA were sequenced to analyze the species diversity and abundance of the simulated microalgae communities at generic level. The accuracy of the sequence results was evaluated, and relationship between the cells counts and the corresponding *actin* gene sequences were established. Simulated samples with a few microalgae cells were also constructed. Both genes were sequenced after whole genome amplification (WGA) treatment to check the feasibility during the application that if only limited organism were provided. The aims of this study were to provide basic data as for the accuracy and limitation of both *actin* and 18S v9 gene in quantitative analysis of phytoplankton community via high-throughput sequencing, and lay foundation for possible application of the technique in field studies.

## Results

### Primary data statistic and quality control

The information as for the process of raw data and its quality control were listed in Additional information Supplement Table 1. Both Miseq sequencing of the *actin* and 18S v9 samples got large amount of data. The value of Q20 and Q30 were greater than 98 and the effective sequences percent were higher than 99% of each sample, which both proved that the sequencing quality was high. The average length of the *actin* sequences and 18S v9 respectively was 206 nt and 134/135 nt, respectively, which proved that the length of amplified *actin* and 18S v9 sequences were stable.

### Generic composition of the *actin* and 18S v9 sequencing samples

#### Generic composition of the *actin* sequencing samples

**Figure 1:**
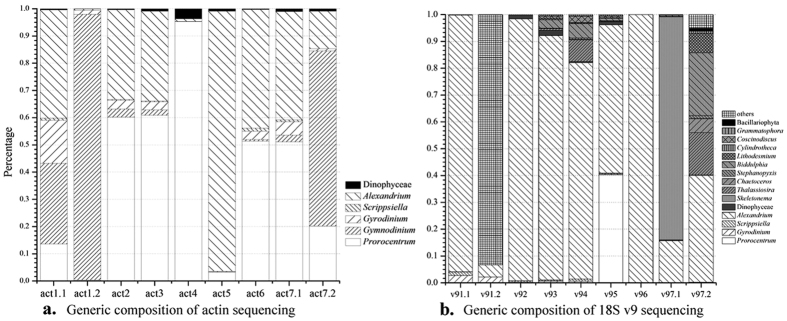
Generic composition of the 9 samples for the *actin* (**a**) and 18S v9 (**b**) sequencing.

The *actin* gene primer pair was designed only for dinoflagellates. The result also showed that the primer worked well and no amplification from diatom was generated. According to our result, dinoflagellates may be classified by *actin* gene at the generic level, thus Miseq data were classified at the generic instead of species level. Sequence composition deduced from Miseq result were showed in Fig. 1a. All the *actin* sequences of dinoflagellates were detected in the act samples; however *Scrippsiella actin* gene was undetectable from sample act4. In addition, a few sequences unable to be annotated to dinoflagellate species existed in all *actin* samples. In sample act1.1, sequences of *Alexandrium* and *Gymnodinium* were dominant (39.79% and 29.622%, respectively); *Prorocentrum* and *Gyrodinium* were posterior (13.613% and 15.825%, respectively) and *Scrippsiella* was the least (only 0.713%). Samples act2 and act3, act6 and act7.1 have similar sequence composition: *Prorocentrum* and *Alexandrium* were dominant, and *Gyrodinium*, *Gymnodinium* and *Scrippsiella* were fewer. Samples act4 and act5 were simulated dinoflagellates samples which imitated *Prorocentrum donghaiense* and *Alexandrium catenella* blooming, respectively, while sequences of *Prorocentrum* and *Alexandrium* dominated the most proportion in sample act4 and act5, respectively. Sequences of *Gymnodinium* in sample act1.2 and act7.2 dominated the larger portion while in the other samples sequences of *Prorocentrum* and *Alexandrium* occupied the large portion and *Gyrodinium*, *Gymnodinium* and *Scrippsiella* only occupied a small part by comparing Fig. 1a and Supplement Table 2.

It was obvious that sequence percentage of the dinoflagellates in the *actin* samples was not completely consistent with the dinoflagellates percentage of cell samples. There was similar deviation when amplifying *actin* gene in different samples. It was especially severe after the whole genome amplified, especially for the *Gymnodinium*. In the simulated blooming samples, sequences of the blooming dinoflagellates were absolutely the majority just similar to their cellular component. The absolute dinoflagellates cell number in samples act2, act3 and act6 were identity while sequence proportion of the dinoflagellates in act6 was not completely the same with those in act2 and act3, which might due to the different portions of dinoflagellates DNA in the total DNA of each sample.

#### Generic composition of the 18S v9 sequencing samples

The community structure of each 18S v9 sample as reflected by gene sequencing was intuitively showed in Fig. 1b. 18S v9 sequences of *Alexandrium* were the majority in sample v91.1 (95.7%), v92 (97.6%), v93 (91.1%), v94 (80.7%) and v96 (100%). Sequences of *Prorocentrum* (40.3%) and *Alexandrium* (55.3%) dominated the sample v95. Sequences of *Skeletonema* were dominant for sample v97.1. In sample v91.2 the majority sequences (93.3%) was neither belonging to dinoflagellates nor diatoms. Species detected in sample v97.2 were much more, and the distribution of sequences was more in accord with their real constitution than the other samples. And it’s worth noting that a few sequences of fungi and bacteria were detected in all 18S v9 samples. The WGA samples v91.2 and v97.2 have large difference in detected species number compared with the corresponding samples v91.1 and v97.1.

In addition, it could be found by comparing Fig. 1b with Supplement Table 2 that sequence percent in all the v9 samples were not consistent with the corresponding cell percent. Even the dinoflagellates or diatoms blooming samples were unable to be reflected by 18S v9 sequencing.

### Amplification deviation of different genus for the *actin* and 18S v9 sequencing

#### Amplification deviation of different genus for the *actin* sequencing

**Figure 2:**
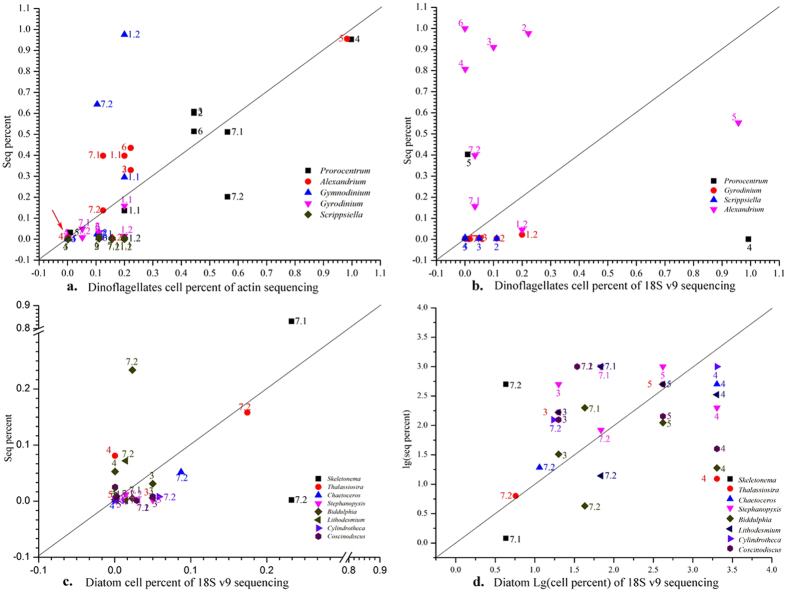
Percent compositions of the sequences and the cells in the 9 samples for the actin (**a**) and 18S v9 (**b–d**) (numbers next to the scatter plots are the number of the samples).

The scatter diagram (Fig. 2a), which showed the ratio of sequence percent to cell percent of each genus, was used to intuitively display the amplification deviation in *actin* samples. All the scatter dots of the samples mainly near to the diagonal except those of samples act1.2 and act7.2, which illustrated that the *actin* amplification bias was enhanced after the WGA. Most of the scatter dots of *Alexandrium* were above on the diagonal and those of *Gyrodinium*, *Gymnodinium* and *Scrippsiella* were under the diagonal. The *actin* seq percent was larger than the real cell percent of *Alexandrium* implied that the amplification bias of *Alexandrium actin* gene was positive; while that of *Gyrodinium*, *Gymnodinium* and *Scrippsiella* were smaller implied that the bias of *Gyrodinium*, *Gymnodinium* and *Scrippsiella* were negative.

The amplification deviation was calculated according to the formula in the portion of “Species composition and community structure analysis of the samples”; while samples act1.2 and act7.2 were excluded considering the serious influence of WGA on the sequence percent. Table 1 showed the detail of the ratio of seq to cell percent, the deviation and the SD. The range of those values for each genus was small and stable. The deviation of *Alexandrium* and *Prorocentrum* was positive. The deviation of *Gymnodinium*, *Gyrodinium* and *Scrippsiella* was negative. And the deviation result was consistent with that showed in the scatter diagram (Fig. 2a).

#### Amplification deviation of different genus for the 18S v9 sequencing

Scatter diagram (Fig. 2b,c) was also used to show the ratio of sequence percent to cell percent of each genus in 18S v9 samples. Dots of the dinoflagellates were scattered and far away from the diagonal, especially the *Alexandrium*, which implied that the sequence percent greatly differed to the corresponding cell percent for dinoflagellates. Although scatters of diatoms were near to the diagonal, the ratios of sequence percent and cell percent for diatoms all were still very large due to the small values of sequence percent and cell percent (such as that of *Scrippsiella* were 0.081 and 0.000495, respectively), which means that the amplification of the diatom 18S v9 deviated largely to their real cell percent.

Thus the scatter diagram of diatoms was reconstructed (Fig. 2d) using the LOG10 value of seq percent and cell percent, and those related dots for most of the samples were still far away from the diagonal. It might imply that the amplification deviation of 18S v9 for dinoflagellates and diatoms were very large. These deviations were calculated and showed in Table 1. As showed in Table 1, some of the deviation, SD and the ratio of sequence percent and cell percent for 18S v9 were large, such as that of *Alexandrium*, *Thalassiosira* and *Biddulphia*, which suggested that 18S rDNA copy number in different cells of these species differed greatly which result in the large deviation of the amplification.

### The diversity of the *actin* and 18S v9 samples

#### Alpha-diversity

**Figure 3:**
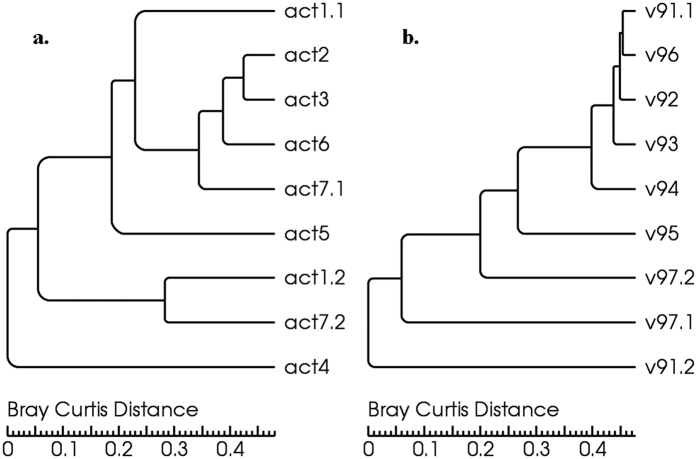
The Rarefaction Curve of the actin (**a**) and 18S v9 (**b**) samples.

The α-diversity analysis was operated according to the sequencing number and the number of OTUs. One OTU was set as a generalized species which was different from the traditional species when doing the α-diversity analysis. The Rarefaction Curve of the *actin* samples was showed in Fig. 3a, the observed species number trend to be stable if more sequences were included, which implied that the sequencing was efficient and only few OTUs would be generated even if more sequencing be operated.

Statistics of the OTUs number and α-diversity, indexes of Chao1 and Shannon were carried out under the sequences similarity of 97% and 95%, respectively to survey the species abundance and composition structure. Details were showed in Supplement Table 3. The OTUs number and indexes of α-diversity under 97% and 95% similarity were mainly consistent. OTUs number of sample act1.2 and act7.2 was fewer and the α-diversity was lower than other samples, especially lower than samples act1.1 and act7.1, respectively. OTUs number and the diversity of act2, act3, act6 and act7.1 were higher than others, and that of act1.1, act4 and act5 were following.α**-**diversity analysis based on OTU analysis was consistent with the species composition. So indexes of alpha diversity of *actin* gene sequencing may be better reflection of the community diversity.

The Rarefaction Curve of the 18S v9 samples was showed in Fig. 3b, species number also trend to be stable if more sequences were involved in the analysis, which implied that the sequencing data got saturated and only few OTUs would be generated even if more sequencing be operated. Statistics of the OTUs number and α-diversity (Chao1 and Shannon) were carried out under the sequences similarity of 97% and 95% to survey the species abundance and composition structure. Details were showed in Supplement Table 3. The OTUs number and Chao1 of the same sample respectively under 97% and 95% similarity were consistent more or less, however, α-diversity indices of the same sample were not consistent. Chao 1 of v97.1 was much higher than other samples while OTUs number and Shannon was close to the others. Each α-diversity indices of sample v93, v94 and v95 were similar, respectively. The OTUs number of WGA samples was less than other samples, while Chao1 of v97.2 was obviously lower and the Shannon were the highest.

#### Beta-diversity

**Figure 4:**
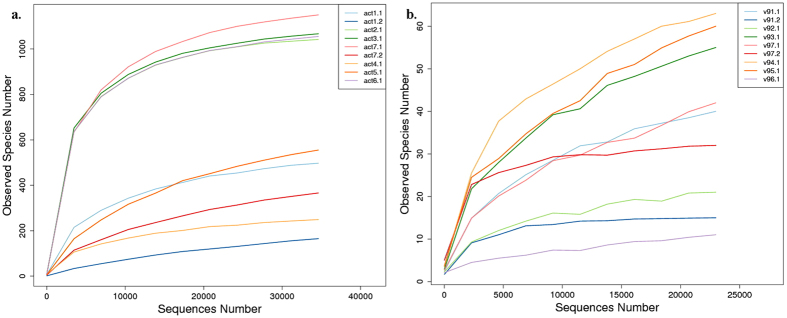
The UPGMA Bray-Curtis tree of the actin (**a**) and 18S v9 (**b**) sequencing samples.

Beta-diversity was showed by the UPGMA Bray-Curtis tree of the act samples (Fig. 4a). Sample act2, act3, act6 and act7.1 firstly clustered and then with act1.1 and act5, while act1.2 and act7.2 clustered together, which implied that act2, act3, act6 and act7.1 have more similar community structure. For the cell-mixed samples, Sample 2, 3 and 6 have the identical dinoflagellates composition, Sample 7 (7.1 and 7.2) has the same dinoflagellates species and different composition with the formers; and Sample 1 (1.1 and 1.2) also has similar dinoflagellates composition with the three formers. *Prorocentrum* and *Alexandrium* respectively dominated the Sample 4 and 5.

It was concluded that the samples having identical or similar dinoflagellates composition clustered together and was distinguishable with blooming simulating samples. Moreover, sequence composition of sample act1.2 and act7.2 differed greatly from those of sample act1.1 and act7.1, and differed greatly from the cell component of samples 1 and 7. Thus, β-diversity analysis of the high-through sequencing of the *actin* gene could cluster the dinoflagellates mixed samples.

B-diversity was showed by the UPGMA Bray-Curtis tree of the 18S v9 samples (Fig. 4b). Sample v91.1, v96, v92, v93, v94, v95, v97.2, v97.1 and v91.2 clustered sequentially, which implied their similarity from strong to weak. However, the clustering relationship of 18S v9 samples was not consistent with their community similarity (the cell percentage of the dinoflagellates and diatoms). The result above showed that the amplification and sequencing of 18S v9 could be not proper for clustering the mixed samples of dinoflagellates and diatoms.

## Discussions

rDNA was the most commonly used genetic marker for the survey of microorganism and phytoplankton in marine environmental community; and 18S rDNA was the first choice of the eukaryotic microbial community survey till now, especially the high variable part, v4 and v9 regions. It was easier to be clustered of 18S v9 sequences, because its short length and consistency, thus may display the eukaryotic community structure and components^1^,^9^,^10^. Yu^2^ evaluated the accuracy of species annotation by using 18S v9 gene and found that result of 16 species annotation by using v9 region was the same as the complete sequence of 18S. Thus, 18S v9 has been chosen to do the analysis of rDNA amplification and Miseq sequencing to evaluate the accuracy of microalgae community analysis.

There were total 20 species belonging to 16 genera exploited in the study. Not all the species were detected in all the 18S v9 samples, and in three blooming samples non-superiority species possessed higher sequences abundance. Result of ITS rDNA clone library^11^ showed that species with higher cell proportion may not be preferentially amplified, while species with lower abundance might get higher amplification efficiency. Kermarrec *et al*.^3^ respectively sequenced the SSU amplified from total DNA of cell mixed sample of different diatom species and SSU DNA mixed sample of the diatoms (equals to the corresponding cell portion of each diatom) by 454 sequencing. They found five species (including three of them with high cell abundance) were not detected from cell mixed sample, while only one species was undetectable for DNA mixed sample, which suggested that PCR reactions of the rDNA templates were independent of cell percentage. Moreover, the OTUs classified from 18S v9 sequences could not cluster the samples corresponding to their phylogenic affiliations. Thus, it could not reflect the actual community structure and constitution of both dinoflagellates and diatoms by the method of rDNA sequencing based on PCR.

In addition, there was some fungal sequence contamination in each 18S v9 sample especially that fungal contamination of sample v91.2 was account for 93.3%. There might be some contaminations in the cultivations and the 18S rDNA of the eukaryotes was conserved. Thus, the amplification of rDNA sequence of fungi by universal primers might exert negative influence on the amplification efficiency for phytoplankton, further increasing the error for evaluating the microalgae community.

The degenerate primer for *actin* gene was designed according to the *actin* sequences of dinoflagellates on NCBI, and the sequences of the *actin* sequencing samples were annotated to dinoflagellates, which showed that the *actin* degenerate primer possessed good specificity aiming at dinoflagellates. *Actin* gene displayed advantages for community survey of the dinoflagellates samples: on one hand, almost all of the genera of dinoflagellates were detected in the actin samples; on the other hand, blooming species got good reflection. In addition, the sequence percent and the cell percent of the same genus was well matched in different *actin* samples, which suggested that the amplification deviation of *actin* gene aiming at dinoflagellates was small and stable and the OTUs classified according to the *actin* gene could accurately reflect the similarity between the dinoflagellates community. It was suggested that it may be a better solution to combine the *actin* sequence percentage from environmental samples with the amplification deviation estimation to elucidate the community structure of environmental samples.

The copy number of *actin* gene in dinoflagellates was still unavailable at present, except that of *Oxyrrhis marina* which was about 3.7^8^. During the process to establish SRD of *actin*, upmost 24 different *actin* subtypes for one species was deposited, which suggested that the *actin* copy for that species was at least 24. Considering that there are highly variations in different *actin* copies within one species^12^,^13^,^14^, it was not unexpected that some *actin* copy type was not included in the sequence dataset, thus unable to be annotated to genus. In the study of Kermarrec *et al*.^3^, taxon outside sequence reference dataset (SRD) were annotated when sequencing the SSU rDNA, *rbcL* and *cox1* amplicons of diatom environmental samples by 454 sequencing.

Compared to 18S v9, the amplification length of *actin* gene was 250 bp, which displayed relative amplification stable among different species due to its protein codoning trait. This made *actin* gene be suitable for the Miseq sequencing and convenient to the pretreatments such as quality filter of the sequencing data. The community structure evaluation based on 18S v9 sequencing differed greatly with the real species abundance, especially when treating blooming samples. ITS rDNA was not suitable for both the dinoflagellates and the diatom samples. The huge difference of rDNA copy number in different kind of species caused the amplification deviation of the sequences (either absent of high abundance species or the exaggeration of low abundance species). Darby *et al*.^15^ analyzed the community structure of the nematode in the soil samples, and corrected the influence of the rDNA copy number to the analysis of sample community; however, this correction was just amended to the classification level on the family.

*Actin* gene amplification and sequencing might be used to analyze the community structure of the microalgae due to the small variation of its copy number in different species. *Actin* gene sequencing was more accurate to describe the proportion of each dinoflagellate species than 18S v9 sequencing, and the amplification deviation of each dinoflagellate genus was obtained based on the *actin* sequencing analysis of the simulated samples.

It could get more OTUs under the same similarity threshold from *actin* sequencing via comparing the sample diversity of the two data sets, which also reflected that the variation of *actin* sequences was very large. Universal primers aimed at *actin* gene have higher specificity, and excluded the contamination from other organism. On the contrary, the amplification of 18S v9 gene easily got contaminated due to its high conservation in eukaryotes, and the clustering relationship of the 18S v9 samples differed greatly with the real sample similarity. Thus, the *actin* gene was thought to be more proper as the molecular marker for analysis of dinoflagellates community in environmental system. Moreover, it would be more precise to show the whole eukaryotic community compositions if more primers aimed at the *actin* gene of more groups was developed.

Comparing the sample act1.2 and act7.2 (WGA samples) with act1.1 and act7.1, species annotated in these samples were the same but their abundance differed greatly, while in the case of 18S v9 sequencing both species attribution and species abundance differed greatly between WGA sample and non-WGA sample. Therefore, DNA content of different species in each sample might get changed after the WGA, this also meant that DNA of different species did not get paralleled amplification during WGA process. Researches have proved that some factors could affect the WGA and generate deviation, such as unbroken genome DNA, extreme GC%, and damage from chemical reagent (like formalin)^16^. As samples undertaken Lugol’s fixed, the amplification of their DNA might be greatly affected by Lugol’s solution. Technique of WGA might not be applied to study the species composition and structure of marine microalgae community at present, or perhaps this kind of kits was inadequate.

Based on our results, the high throughput sequencing of *actin* gene could be more precise for the analysis of dinoflagellates community than using rDNA. Therefore, *actin* gene is a potential molecular marker for the community structure analysis of eukaryotic microbial, especially aiming to precisely quantify the species abundance of the eukaryotic community.

## Materials and Methods

### Species and cultivations

Totally 9 dinoflagellate species and 11 diatoms belong to different genera were used in the studies. Lugol’s fixed samples of dinoflagellates species were kindly provided by Research Center for Harmful Algae and Marine Biology, Jinan University, except *Alexandrium catenella*. All the other species were live cultures from Microalgae Culture Collection of Key Laboratory of Marine Genetics and Breeding of Ministry of Education, College of Marine Life Sciences, Ocean University of China. The live cells were maintained in F/2 medium^17^ and incubated at 20 °C under a 12 h:12 h light-dark cycle at low irradiance (10–25 μmol photons·m^−2^·s^−1^). The live cultures of diatom strains were identified to specific or generic level using morphological characters based on observations under light and scanning electron microscopy or transmission electron microscope, then their taxonomic status were confirmed mainly according to the books “Identification marine diatom and dinoflagellate (edited by Carmelo R. Tomas)” and “The Diatom World (edited by Joseph Seckbach and J. Patrick Kociolek)”.

### Preparation of the mixed samples

7 mixed samples were set in this study to simulate different cell components in natural environment. The species composition and the cell percentage of these simulated samples at generic level were showed in Supplement Fig. 1. For the blooming simulated samples, the cell number of the bloom species was according to the low threshold value of “Red tides occurrence criteria” in the State Environmental Protection Standards of *the People’s Republic of China HJ 442–2008 Specification for Offshore Environmental Monitoring*^18^.

### DNA extraction, PCR amplification and sequencing

Cell sediments of the samples were washed twice with 1× TE buffer, and their total DNA were extracted by 2× CTAB^19^, then the purity and concentration of the DNA were checked and diluted to 50 ng/μL. DNA of the Sample 1.1 and 7.1 were not diluted because of the low DNA concentration (about 10 ng/μL). The *actin* gene and 18S v9 of every DNA sample was respectively amplified. The 18S v9 could be amplified from DNA of dinoflagellates and diatoms, and the *actin* gene could be amplified only from DNA of dinoflagellates. There were barcodes added to both 5′-ends of forward and reverse primers, the barcode sequences were showed in Supplement Table 4. Primers of 18S v9 were 18S v9-F (5′-CCCTGCCHTTTGTAC ACAC-3′) and 18S v9-R (5′-CCTTCYGCAGGTTCACCTAC-3′), primers of actin gene were act-F (5′-ATGGTNGGYATGGACCARAA-3′) and act-R (5′-AAVGTCTCRAACATRATYTGNGTCA-3′), and sequence data used as template for the *actin* gene primer design were showed in Supplement Table 5. PCR cycling conditions were one cycle of 94 °C 10 min, then 35 cycles of 94 °C 40 s for v9 (1 min for *actin*), 58 °C 25 s for v9 (30 s for *actin*) and 72 °C 30 s for v9 (40 s for *actin*) followed by a final extension of 72 °C 10 min. Five replicates were run for each reaction and the five amplicons were pooled on a 1.5% agarose gel. Target product (~180 bp/~250 bp) of each mixed sample was excised and recovered by GIAquick GEL Extraction kit (QIAGEN, American). PCR-free library was constructed by using NEB Next^®^ Ultra™ DNA Library Prep Kit for Illumina (New England Biolabs, American). Miseq sequencing was carried out by Beijing Novogene biological information technology company, LTD.

### Whole genome amplification

The total DNA of sample 1.2 and 7.2 were isolated and amplified by QIAGEN REPLI-g Single Cell Kit (QIAGEN, American) according to the kit operation manual to get whole genome amplified. Only 10–15 ng total DNA was used as the initial template for the WGA, and then the product was used as the template of the following PCR, gel recovery and Miseq sequencing for sample 1.2 and 7.2.

### SRD preparation for species annotation

For the preparation of SRD of *actin* gene, sequences of *actin* were amplified by the primer pair act-F/R, then cloned into PMD18-T vector (Takara, Japan) and 20 clones of each dinoflagellate species were sequenced for *actin* gene to construct the dataset. For the 18S v9 library, 18S v9 of all the dinoflagellates and diatoms species was respectively amplified and sequenced, then added to the Silva Database (http://www.arb-silva.de/) as the 18S v9 SRD for species annotation.

### Miseq sequencing data analysis

Paired-end reads assemblies: Paired-end reads were assigned to each sample based on their unique barcode and truncated by cutting of the barcode and primer sequence; after initial trimming we obtained the merged reads with FLASH v1.2.7 (http://ccb.jhu.edu/sofware/FLASH/)^20^ based on overlapping regions within paired-end reads, and the splicing sequences were called raw tags; qualities filtering on the raw tags were performed according to the QIIME v1.7.0 (http://qiime.org/index.html)^21^ quality control process; and we obtained the high-quality clean tags (Effective tags) after that the chimaera sequences were removed through the UCHIME algorithm (http://www.drive5.com/usearch/manual/uchime_algo.html)^22^. OTU cluster: We use Uparse v7.0.1001 (http://drive5.com/uparse/)^23^ to pick operational taxonomic units (OTUs) by making OTU Table. sequences with ≥97% similarity were assigned to the same OTUs. Species annotation: For each OTU, a representative sequence was screened and used to assign taxonomic composition using the prepared SRD of actin gene and 18S rDNA (cutoff was set to 0.8–1), then the taxon abundance of each sample was generated into phylum, class, order, family, and genera levels. Statistical analysis: Metastats software was utilized to confirm differences in the abundances of individual taxonomy between the two groups, LDA EffectSize (LEfSe) was used for the quantitative analysis of biomarkers within different groups; then all of the analyses from clustering to alpha (within sample) and beta diversity (between samples) were performed with QIIME (v1.7.0) and displayed with R software (v2.15.3).

### Species composition and community structure analysis

The sequences of each sample were respectively classified at the generic level to know the species composition of each sample according to the sequencing results of *actin* and 18S v9 gene. The correlation (amplification deviation) between sequences percentage and cell percentage of the species was calculated according to the formula below, which may reflect the increasing or decreasing amplification degree.


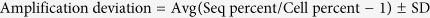


### Species diversity analysis

The analysis of species diversity was based on the sequences diversity of the OTUs clusters. Rarefaction curves, species richness estimators and community diversity indices were used to indicate the species richness and community diversity and show the α-diversity of the samples. And Chao1^24^ and Shannon^25^ were the specific indexes of species richness estimators and community diversity indices. B-diversity showed the comparison of community composition between samples, and it was measured by the calculation of Bray-Curtis distance^26^,^27^ and then displayed by UPGMA analysis.

## Additional Information

**How to cite this article**: Guo, L. *et al*. Quantitative analysis of dinoflagellates and diatoms community via Miseq sequencing of *actin* gene and v9 region of 18S rDNA. *Sci. Rep.* **6**, 34709; doi: 10.1038/srep34709 (2016).

## Supplementary Material

Supplementary Information

## Figures and Tables

**Table 1:** The deviation and sequence percent/cell percent of the genera for *actin* and 18S v9 sequencing.

Genus	seq percent/cell percent	Deviation	SD
For *actin* sequencing samples			
* Prorocentrum*	0.6806–3.3078	0.39	±0.8811
* Alexandrium*	0.9718–3.1823	0.682	±0.8272
* Gymnodinium*	0.0133–1.4811	−0.58	±0.4851
* Gyrodinium*	0.069–1.5656	−0.316	±0.5144
* Scrippsiella*	0.0218–0.0707	−0.963	±0.019
For 18S v9 sequencing samples			
* Prorocentrum*	0.001~42.214	13.073	±24.3707
* Gyrodinium*	0.036~10.095	0.497	±3.4977
* Scrippsiella*	0.036~16.152	2.519	±7.081
* Alexandrium*	−0.775~10008.5	1205.571	±3311.9429
* Skeletonema*	0.0086~3.5776	0.7931	±2.5237
* Thalassiosira*	0.12~163.539	40.351	±81.4596
* Chaetoceros*	0.596~4.038	1.317	±2.4337
* Stephanopyxis*	0.04~10.095	1.29	±4.3749
* Biddulphia*	−0.785~106.007	23.335	±46.3827
* Lithodesmium*	−0.9312~5.057	1.407	±2.8708
* Cylindrotheca*	0.1376~2.019	0.0783	±1.3304
* Coscinodiscus*	−0.9656~49.475	9.727	±22.2541
