# Supplementary material for: Quantitative analysis of dinoflagellates and diatoms community via Miseq sequencing of actin gene and v9 region of 18S rDNA
Source: Sci Rep. 2016 Oct 10;6:34709. doi: 10.1038/srep34709 (PMC5056376; doi:10.1038/srep34709)
Supplement: Supplementary Information [file srep34709-s1.pdf]

Title.

Quantitative analysis of dinoflagellates and diatoms community via Miseq sequencing of *actin* gene and v9 region of 18S rDNA

Author list and affiliations.

Guo Liliang[1, 2], gll1224@126.com

Sui Zhenghong[1]*, suizhengh@ouc.edu.cn

Liu Yuan[1], liuyuan_1991@yeah.net

[1]Key Laboratory of Marine Genetics and Breeding of Ministry of Education, College of Marine Life Sciences, Ocean University of China, Qingdao 266003, China.

Address. 5 Yushan Road, Qingdao 266003, Shandong, China.

[2]Institution of Plastic Surgery, Weifang Medical University, Weifang, 261042, China.

Address. 4948 Shengli East Street, Weifang 261042, Shandong, China.

***Corresponding author.**

Sui Zhenghong, suizhengh@ouc.edu.cn, +86-0532-82031128

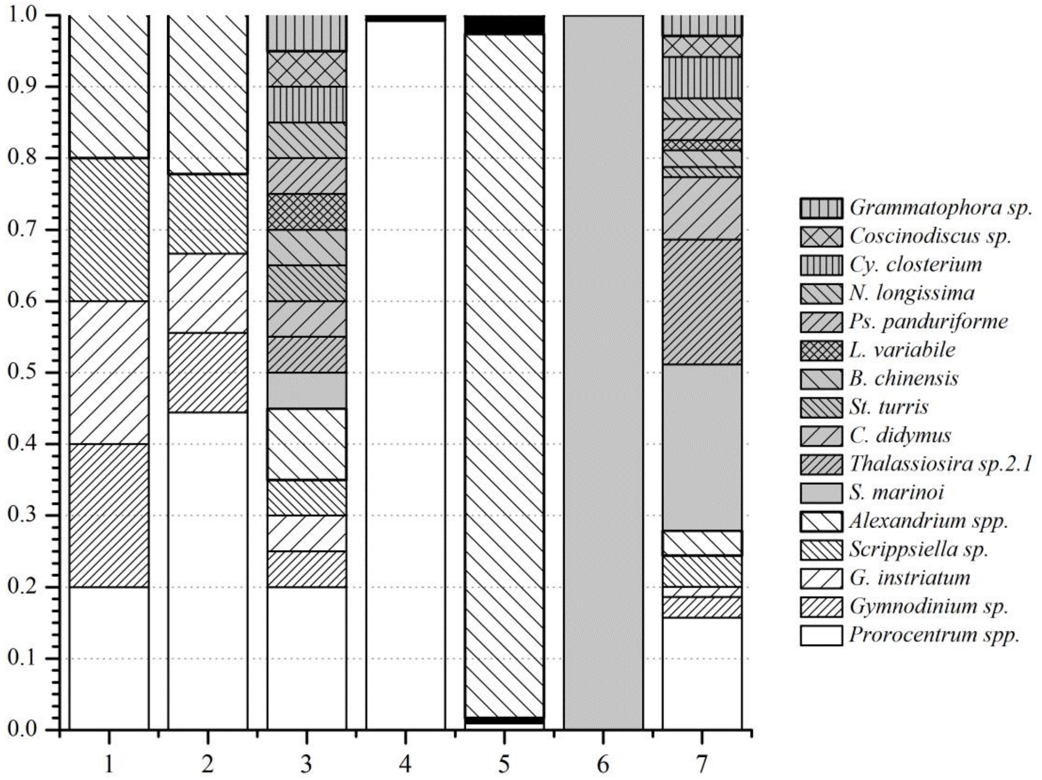

Supplement Fig. 1 Species composition and cell percentage of 7 simulated samples at generic level. Many species just possessed tiny proportion brought out the black bar in column 4, 5 and 6.

Supplement table 1 Statistics on the raw sequence data process and quality control for the *actin* and 18S v9 Miseq sequencing

Sample	Raw PE (#)	Raw Tags (#)	Effective Tags (#)	Base (nt)	AvgLen (nt)	Q20	Q30	GC%	Effective-P (%)
act1.1	46850	46516	46514	9570573	206	98.83	98.52	59.42	99.28
act1.2	88642	87855	87847	18098101	206	98.63	98.29	61.11	99.10
act2	46396	46075	46071	9482782	206	98.70	98.31	61.29	99.30
act3	42649	42274	42269	8700308	206	98.73	98.37	61.29	99.11
act4	37535	37357	37353	7703474	206	98.59	98.18	61.98	99.52
act5	44974	44687	44684	9194272	206	98.80	98.50	58.22	99.36
act6	50393	49999	49987	10291238	206	98.63	98.21	61.06	99.19
act7.1	80407	79788	79787	16421574	206	98.74	98.35	60.69	99.23
act7.2	58849	58552	58550	12062858	206	98.63	98.28	60.01	99.49
v91.1	23140	23098	23093	3094436	134	99.51	99.15	43.72	99.80
v91.2	83354	82930	82917	11040412	133	99.44	99.07	47.36	99.48
v92	27490	27432	27427	3675255	134	99.47	99.11	43.54	99.77
v93	33209	33056	33045	4428345	134	99.41	98.98	44.07	99.51
v94	30146	30025	30015	4023594	134	99.48	99.12	45.05	99.57
v95	41153	40972	40963	5488512	134	99.47	99.06	47.02	99.54
v96	54423	54330	54320	7278797	134	99.45	99.07	43.34	99.81
v97.1	80614	80223	80204	10814006	135	99.28	98.80	48.72	99.49

v97.2	37385	37221	37209	5010228	135	99.35	98.83	48.33	99.53

Q20 and Q30 showed the percentage of Effective Tags that the base quality was more than 20 (sequencing error rate < 1%) and 30 (sequencing error rate < 1%); GC% showed the GC content in Effective Tags; Effective-P (%) showed the ratio of Effective Tags to Raw PE reads.

Supplement table 2 Constituent of seven samples and their cell number

Species	Cells of the species in each sample						
	1.1/1.2	2	3	4	5	6	7.1/7.2
Dinoflagellate							
Prorocentrum donghaiense	500	500	500	1000000	500	500	400
Prorocentrum minimum	0	500	500	500	500	500	100
Prorocentrum micans	0	500	500	500	500	500	20
Prorocentrum sigmoides	0	500	500	500	500	500	20
Scrippsiella sp.	500	500	500	500	500	500	150
Gymnodinium sp.	500	500	500	500	500	500	100
Gyrodinium instriatum	500	500	500	500	500	500	50
Alexandrium sp.	0	500	500	500	500	500	20
Alexandrium catenella	500	500	500	500	200000	500	100
Diatom							
Skeletonema marinoi	0	0	500	500	500	10000000	800
Thalassiosira sp. 2.1	0	0	500	500	500	500	600
Chaetoceros didymus	0	0	500	500	500	500	300
Stephanopyxis turris	0	0	500	500	500	500	50
Biddulphia chinensis	0	0	500	500	500	500	80
Lithodesmium variabile	0	0	500	500	500	500	50
Psammodictyon panduriforme	0	0	500	500	500	500	100
Nitzschia longissima	0	0	500	500	500	500	100
Cylindrotheca closterium	0	0	500	500	500	500	200
Coscinodiscus sp.	0	0	500	500	500	500	100
Grammatophora sp.	0	0	500	500	500	500	100

Equal amount of dinoflagellates cells from different genera were mixed in sample 1 to evaluate the amplification efficiency of the primers in different dinoflagellates genera; sample 2 contained equal amount of cells of different dinoflagellates species to simulate the dinoflagellate water system; sample 3 contained more equal amount of cells of different diatoms species based on the containing of sample 2. Sample 4, 5 and 6 imitated the bloom of *P. donghaiense*, *A. catenella* and *S. marinoi*, respectively. It was unequal amount of cells of different dinoflagellates and trace diatom cells in sample 7. Sample 1 and 7 were divided into two equal parts (1.1 and 1.2, 7.1 and 7.2); sample 1.2 and 7.2 were used to do the WGA after total DNA extraction.

Supplement table 3 Alpha diversity indices for the *actin* samples and 18S v9 samples

samples	OTU (97%)	OTU (95%)	Chao1 (97%)	Chao1 (95%)	Shannon (97%)	Shannon (95%)
act1.1	497.0	321.0	540.925	353.571	4.864	4.309
act1.2	165.0	123.0	355.778	154.784	0.291	0.294
act2	1042.0	572.0	1065.697	581.091	7.870	6.873
act3	1067.0	595.0	1137.040	620.625	7.945	6.925
act4	249.0	162.0	265.776	184.885	4.717	4.158
act5	555.0	370.0	662.897	412.533	4.470	3.582
act6	1055.0	581.0	1106.562	589.724	7.718	6.664
act7.1	1150.0	638.0	1217.391	652.170	7.458	6.601
act7.2	366.0	285.0	507.359	415.784	2.755	2.531
v91.1	40.0	33.0	44.583	49.500	1.241	0.335
v91.2	15.0	14.0	15.000	19.000	0.580	0.501
v92	21.0	14.0	22.429	15.500	1.133	0.204
v93	55.0	39.0	65.200	54.600	1.569	0.702
v94	63.0	45.0	76.333	64.429	1.996	1.201
v95	60.0	35.0	89.545	46.000	1.918	1.335
v96	11.0	6.0	14.333	9.000	0.953	0.004
v97.1	42.0	33.0	105.250	223.000	0.921	0.761
v97.2	32.0	20.0	33.000	20.000	2.868	2.419

Supplement table 4 Sequences of the primer barcode for each sample

Sequencing sample	Primer name	Barcode	Sequencing sample	Primer name	Barcode
act1.1	act-1F	CGATGT	v91.1	18-1F	CTTGTA
	act-1R	GGTAGC		18-1R	CATGGC
act1.2	act-8F	ATCACG	v91.2	18-8F	GTGAAA
	act-8R	GAGTGG		18-8R	GACGAC
act2	act-2F	TGACCA	v92	18-2F	AGTCAA
	act-2R	ATGAGC		18-2R	CATTTT
act3	act-3F	GATCAG	v93	18-3F	AGTTCC
	act-3R	CACGAT		18-3R	CCAACA
act4	act-4F	TTAGGC	v94	18-4F	ATGTCA
	act-4R	ACTGAT		18-4R	CGGAAT
act5	act-5F	CAGATC	v95	18-5F	CCGTCC
	act-5R	CAACTA		18-5R	CTAGCT
act6	act-6F	ACTTGA	v96	18-6F	GTAGAG
	act-6R	CACCGG		18-6R	CTATAC
act7.1	act-7F	GGCTAC	v97.1	18-7F	GTCCGC
	act-7R	CAGGCG		18-7R	CTCAGA
act7.2	act-9F	ACAGTG	v97.2	18-9F	GTGGC
	act-9R	ATTCCT		18-9R	TAATCG

Supplemental table 5 Sequence data used as template for the *actin* gene primer design

GenBank No.	Species	Taxonomic status
DQ174766	*Symbiodinium* sp.	Dinophyceae; Suessiales; Symbiodiniaceae
AB231900	*Symbiodinium* sp.	Dinophyceae; Suessiales; Symbiodiniaceae
DQ867056	*Karlodinium micrum*	Dinophyceae; Gymnodiniales; Kareniaceae
EF134084	*Karlodinium micrum*	Dinophyceae; Gymnodiniales; Kareniaceae
JX262505	*Lepidodinium chlorophorum*	Dinophyceae; Gymnodiniales; Gymnodiniaceae
EF134225	*Noctiluca scintillans*	Dinophyceae; Noctilucales; Noctilucaceae
EU742739	*Amphidinium carterae*	Dinophyceae; Gymnodiniales; Gymnodiniaceae
EU742748	*Amphidinium carterae*	Dinophyceae; Gymnodiniales; Gymnodiniaceae
JX262499	*Adenoides eludens*	Dinophyceae; Gonyaulacales; Gonyaulacaceae
JX262503	*Azadinium spinosum*	Dinophyceae; Gonyaulacales; Amphidomataceae;
HQ391374	*Dinophysis acuminata*	Dinophyceae; Dinophysiales; Dinophysiaceae
HQ391397	*Dinophysis caudata*	Dinophyceae; Dinophysiales; Dinophysiaceae
JX262509	*Thecadinium kofoidii*	Dinophyceae; Peridiniales; Oxytoxaceae
AY423582	*Lingulodinium polyedrum*	Dinophyceae; Gonyaulacales
JX262504	*Gymnodinium aureolum*	Dinophyceae; Gymnodiniales; Gymnodiniaceae
JX262506	*Pentapharsodinium dalei*	Dinophyceae; Peridiniales; Peridiniaceae
AF421536	*Crypthecodinium cohnii*	Dinophyceae; Gonyaulacales; Crypthecodiniaceae
EF133869	*Alexandrium fundyense*	Dinophyceae; Gonyaulacales; Gonyaulacaceae
JX262500	*Alexandrium fundyense*	Dinophyceae; Gonyaulacales; Gonyaulacaceae
JX262501	*Alexandrium minutum*	Dinophyceae; Gonyaulacales; Gonyaulacaceae
AB231901	*Symbiodinium sp.*	Dinophyceae; Suessiales; Symbiodiniaceae
